# Supplementary material for: Economic evaluation of anti-malarial drug policies across presidential regimes in Nigeria: A comparative analysis from 1999 to present
Source: PLoS One. 2026 Mar 11;21(3):e0344909. doi: 10.1371/journal.pone.0344909 (PMC12978454; doi:10.1371/journal.pone.0344909)
Supplement: Attachment — Submitted filename: review_PONE-D-24-42404-1.pdf [file pone.0344909.s001.pdf]

Review of Manuscript No PONE-D-24-42404

Title: Economic Evaluation of Anti-Malarial Drug Policies Across Presidential Regimes in Nigeria: A Comparative Analysis from Obasanjo, Yar'Adua, Jonathan, Buhari, to Tinubu

Article Type: Research Article

Keywords: Malaria; economic evaluation; Anti-malarial Policies; Nigeria; cost-effectiveness; Public health

1 General Comments

The article is addressing an important area of public health policy. Results from studies of the type conducted here are informative for it gives a clear picture on the evolution and performance of the health policies of the chosen country and at the same time evaluates the effectiveness of control measure usually applied by governments. In the case of malaria, many countries in sub-Saharan Africa do have National Malaria Control Programmes (NMCPs) and it will be very useful to conduct evaluations of the type reported in this article. It is important to recognize that the work presented in the manuscript is cost, and labour intensive, and the output are of far reaching importance.

The present paper reports results of a comparative study of policies of five different administrations in the Nigeria. The work therefore covers a long period of time (1999-2023). The study area and materials and methods are clearly outline and stated. Though I did not see in the manuscript the idea of evaluation. The language in the manuscript is clear and void of typos. **The literature review is not in sink with the results reported**:

2 Technical considerations

The manuscript, being as it is, a report from activities of past administrations in Nigeria and comparing the results, we can only examine the report for consistency. To this effect, I make the following observation and then some technical comments: **I made an attempt to examine the manuscript for consistency and form of presentation as well as the results obtained, but my efforts were frustrated with the style and general presentation of the manuscript. I therefore stopped assessing the manuscript for the following reasons:**

1. The entire manuscripts lacks paragraphs, lacks numbering of sections and subsections, lacks punctuations, making it very difficult to follow.

2. The analytic methods alluded to on page 10 are vaguely articulated...the economic evaluation employs several analytical methods...

3. The manuscript has 5 small tables that could easily have been incorporated into the body of the manuscript for easy reading , but these were supplied as supplementary material to be downloaded separately. Once the table are downloaded, one does not see the economic evaluation alluded to in the title. The authors need to define what they mean by evaluation and then tell us what metrics they intend to use to do the evaluation. Is comparing the same as evaluation? What I discern from the manuscript is that the authors have compared the policies of the different administrations.

4. Almost all references were seen not to be supporting the information to which the indicated reference is pointing

at. For example, refs 43-46 have nothing to do with currency, price date and conversion. These wrong associations with citation and cited sources runs through the manuscript Refs 1,2 on page three which was first used at the section where the manuscript is talking about the fact that ...” The World Health Organization (WHO) estimated that in 2022, Nigeria contributed to approximately 27% of global malaria cases and 23%of malaria deaths, making it the country with the highest malaria mortality worldwide [1][2]...”, is the same reference cited twice. Refs 47-51 on the section about choice of model on page 9 say nothing about Markov models.

5. It looks like the references, within the body of the text, are simply listed from 1- 86 without any regard as to whether the are correctly cited, or whether the cited reference is providing more information to the section of text being referenced.

6. Figures are cited within the text but were not substantiated with a labeled figure. Though we found some graphs, one could not tell which graph, is figure 1 or figure 2, etc. For example, Figure 1 is referred in the section about choice of model on page 9, but there is no figure labeled as figure 1. There is a compartmental flow chart with four compartments (Healthy, Infected, Dead, Treated) that has been used to describe transitions in a Markov Model. This is definitely not a good rendition of the idea of a Morkov Model, and more so the transition arrows and labels on the figure are obscured thick black lines making it difficult for anyone to read what is written on the chart.

7. **Several inconsistence:** When it was proving difficult to evaluate the manuscript, I stopped reading it. It is the re-

sponsibility of the authors and editors of the Journal to ensure that reviewers get a document which can enable them speedily evaluate a manuscript.

8. **Caution:** The Journal should check to be certain that this manuscript is not AI generated.

3 Final comments

It is my opinion that this paper is an important piece of work and that the results are very useful for calibrating malaria control programs in Nigeria. However, **I do not recommend the paper for publication in the current form**, unless the major issues pointed above are clarified and a more coherent article presented.
