# Supplementary material for: Economic evaluation of anti-malarial drug policies across presidential regimes in Nigeria: A comparative analysis from 1999 to present
Source: PLoS One. 2026 Mar 11;21(3):e0344909. doi: 10.1371/journal.pone.0344909 (PMC12978454; doi:10.1371/journal.pone.0344909)
Supplement: Attachment — Submitted filename: Reviewer response.docx [file pone.0344909.s002.docx]

Dear Reviewer 1,

Thank you for your valuable feedback and constructive suggestions. We have carefully addressed all your comments and made the necessary revisions to improve the clarity, structure, and analytical rigor of the manuscript.

Regarding your concern about the idea of evaluation, we have explicitly defined economic evaluation in the **Introduction**, clarifying that it involves both **comparative analysis and formal economic modeling** using cost-effectiveness and cost-benefit metrics. We have also detailed the specific evaluation measures, including **cost per HALY gained, incremental cost-effectiveness ratios (ICERs), and total program expenditure relative to malaria morbidity reduction.**

We have revised the **Materials and Methods** section to provide a clearer articulation of the **analytical methods**, including **cost-effectiveness analysis (CEA), cost-benefit analysis (CBA), probabilistic sensitivity analysis (PSA) using Monte Carlo simulations, survival analysis, and meta-analytic techniques.** These revisions ensure a more robust explanation of our approach to evaluating anti-malarial policies.

To enhance readability and organization, we have restructured the manuscript by adding **clear paragraphing, numbered sections and subsections, and appropriate punctuation.** This improves the manuscript’s flow and makes it easier to follow.

Regarding the presentation of tables, we have now **incorporated the five small tables into the main body** of the manuscript instead of providing them as supplementary material. This allows for easier access to key data and enhances the integration of economic evaluation findings within the text.

We have conducted a thorough review of all **citations and references** to ensure that they correctly support the information presented in the manuscript. Incorrectly assigned references (e.g., Refs 43-46, 47-51) have been corrected, and duplicate references (e.g., Refs 1 and 2) have been removed. We have ensured that all cited sources align with the content being referenced.

For **figures and graphs**, we have properly labeled and numbered all figures within the text. The **compartmental flow chart representing the Markov Model has been revised** for better readability, with clear transition arrows and labels. The obscured black lines have been corrected to ensure that all elements are visible and easily interpretable.

We appreciate your insightful feedback, which has significantly strengthened the manuscript. Please let us know if further modifications are needed.

Best regards,
Authors

Dear Reviewer 2,

Thank you for your comprehensive and insightful feedback. We appreciate the depth of your questions and suggestions, which have significantly contributed to strengthening the manuscript. We have carefully addressed each of your concerns and incorporated relevant revisions and discussions throughout the manuscript.

We have considered your suggested title change and, while the original title aligns with our study objectives, we acknowledge the merit of your suggestion and have adjusted it for clarity and broader readability.

To address the issue of **inflation adjustments**, we have included a sensitivity analysis that evaluates how adjusting for inflation across different regimes influences the perceived cost-effectiveness of malaria policies. We discuss whether effectiveness rankings among administrations change as a result.

Regarding the **role of foreign aid**, we have expanded our discussion on the impact of external funding from initiatives such as the **Presidential Malaria Initiative, WHO interventions, and the Gates Foundation.** We now analyze the extent to which reductions in malaria prevalence can be attributed to external funding versus domestic policy efforts.

We have accounted for **population growth** in our cost-effectiveness analysis and discussed how it may influence the sustainability of malaria policies over time. The potential impact of increased population size on **HALYs gained** has also been explicitly addressed.

To reflect **indirect benefits**, we have expanded our discussion on how malaria control interventions may have led to **improved healthcare infrastructure, enhanced malaria diagnostics, and overall healthcare system strengthening**, which could have influenced our findings.

We have also examined **shifts in recommended treatments**, particularly the adoption of **artemisinin-based combination therapies (ACTs)** across different administrations, and how these shifts impact **policy sustainability and long-term effectiveness.**

Regarding **indirect costs**, we have incorporated a discussion on other economic burdens, such as **increased healthcare expenditures, caregiver burden, and opportunity costs** beyond lost productivity, to provide a more comprehensive view of the economic impact of malaria control.

We have expanded our discussion on **drug resistance**, particularly how its emergence could alter the long-term effects predicted by the Markov model and its implications for future malaria policies.

We now include an assessment of **regional differences** in **Intermittent Preventive Treatment in Pregnancy (IPTp)**, particularly how **variations in healthcare access and maternal health education** could affect its effectiveness.

We have discussed how **malaria transmission intensity varies across Nigeria’s ecological zones** and emphasized the need for **region-specific malaria control strategies** that prioritize interventions based on epidemiological data.

To strengthen the reliability of cost comparisons, we have provided additional context on how **potential fluctuations in the Consumer Price Index (CPI) and exchange rate volatility** may impact the study’s results. We explore whether alternative inflation adjustments could yield substantially different findings.

We acknowledge the **challenges in sourcing historical cost and inflation data** and have explicitly stated any limitations related to data gaps. Our approach to adjusting historical costs to a **base year of 2024** has been clarified, along with a discussion of **purchasing power differences over time** and how they might affect economic interpretations.

The sustainability assessment of each policy has been refined to incorporate the influence of **exchange rate fluctuations and inflation adjustments**, revealing **hidden costs or benefits that were not initially apparent.**

To account for the impact of **counterfeit anti-malarial medicines**, we now discuss how their prevalence may have led to **an overestimation of policy effectiveness.** We also explore how strengthening **regulatory oversight** could provide more accurate assessments of intervention outcomes.

We have examined the **cost-benefit trade-offs** of enhancing regulatory oversight and supply chain improvements, analyzing how reallocating resources could impact other critical health programs.

Our discussion now includes an estimate of **projected economic gains in GDP or productivity** if malaria incidence were reduced by **50%**, strengthening the policy argument for increased investment in malaria control.

We have incorporated a section on **long-term cognitive impairments caused by malaria in children under five** and their implications for Nigeria’s future economic growth, particularly in skilled labor sectors. We highlight the potential benefits of **integrating malaria prevention with education and workforce development programs.**

Finally, we have expanded our discussion on the **potential benefits of increased international collaboration with WHO, the Global Fund, and other organizations** to improve the quality assurance of anti-malarial treatments and strengthen the supply chain. We also discuss potential barriers to these collaborations.

We appreciate your valuable feedback, which has greatly improved the clarity and depth of our manuscript. Please let us know if further modifications are required.

Best regards,
Authors

Dear Reviewer 3,

Thank you for your thoughtful comments and valuable suggestions. We have carefully addressed each of your points and made the necessary revisions to enhance the clarity and rigor of our manuscript.

We have expanded our explanation regarding the **fixed 3% discount rate**, detailing why it was appropriate for Nigeria and discussing its relevance in economic evaluations. We have also clarified why this rate remains constant despite economic fluctuations, drawing from established health economic guidelines and comparative studies.

Regarding the assumption of **constant anti-malarial effectiveness**, we acknowledge its potential limitations and have now included a discussion on how this assumption may impact the **generalizability of the study’s findings.** We explore how variations in effectiveness due to factors such as drug adherence, supply chain issues, and treatment resistance could influence cost-effectiveness outcomes.

We have also incorporated a section discussing the **limitations and potential biases of data sourced from the National Health Information Systems and peer-reviewed studies.** We highlight how data inconsistencies, underreporting, and regional disparities could impact the robustness of our findings.

Lastly, we have addressed the impact of **anti-malarial drug resistance** on long-term cost-effectiveness. We discuss how increasing resistance could reduce the efficacy of current treatment strategies, thereby **raising costs and necessitating policy adaptations.** We also explore how proactive resistance monitoring and policy adjustments could mitigate these risks.

Your feedback has greatly improved the manuscript, and we appreciate your time and effort in reviewing our work. Please let us know if further clarifications are needed.

Best regards,
Authors
