# Supplementary material for: Economic evaluation of anti-malarial drug policies across presidential regimes in Nigeria: A comparative analysis from 1999 to present
Source: PLoS One. 2026 Mar 11;21(3):e0344909. doi: 10.1371/journal.pone.0344909 (PMC12978454; doi:10.1371/journal.pone.0344909)
Supplement: Attachment — Submitted filename: Reviewer_response_auresp_2.docx [file pone.0344909.s003.docx]

**Reviewer #1 Comment:**
“I was concerned about the scientific nature of the evaluation, and I am still not satisfied with the scientific evaluation.”

**Authors' Response:**
We sincerely thank reviewer #1 for this important comment regarding the scientific rigor of our evaluation. In response, we have undertaken substantial revisions across multiple sections of the manuscript to strengthen the methodological and analytical framework of the study. The additions and modifications are highlighted in **red color** in the revised version.

1. **Abstract:**
   We have clarified the type of economic evaluation conducted (**cost-effectiveness and cost–benefit analyses**) and explicitly stated the **societal perspective** adopted. Quantitative indicators, including **cost per HALY gained** and **incremental cost-effectiveness ratios (ICERs)**, were added to emphasize methodological rigor.
2. **Introduction:**
   We refined the rationale for comparing anti-malarial drug policies across successive presidential regimes and supported this with additional literature on malaria control economics and policy transitions in Nigeria.
3. **Methods:**
   This section was comprehensively revised to enhance scientific transparency and robustness. Specifically, we:

- Defined the evaluation type (**full CEA and partial CBA**) and **societal perspective**.
- Added details on **time horizon**, **currency adjustments for inflation**, and **exchange rate normalization**.
- Expanded the description of **data sources** and analytical procedures, including **health outcome and cost data extraction**.
- Included a **sensitivity analysis** to evaluate uncertainty in cost and effectiveness parameters.
- Justified the use of successive **political regimes as comparator groups** for policy impact evaluation.
- Cited standard methodological references, including Drummond et al. (2015) and WHO-CHOICE guidelines, to align with internationally recognized frameworks.

4. **Results:**
   We presented the comparative findings more systematically, summarizing **costs, outcomes, and ICER values** by administration. Tables were structured to improve clarity and highlight differences in policy efficiency.
5. **Discussion:**
   The discussion now provides a more rigorous interpretation of results, addressing **model validity**, **contextual limitations**, and **policy implications** within Nigeria’s economic and public health landscape.
6. **References:**
   We updated and reformatted all references in **Vancouver style**, ensuring inclusion of authoritative sources such as Drummond et al. (2015) and WHO-CHOICE.

We trust that these revisions have substantially improved the **scientific quality, transparency, and methodological soundness** of the manuscript.

**Reviewer #2 Comment:**
“All scientific comments are addressed. However, I suggest authors check the guideline of PLOS ONE and format the manuscript accordingly.”

**Authors' Response:**
We thank reviewer #2 for this helpful reminder regarding journal formatting. We have carefully reviewed and implemented the PLOS ONE formatting guidelines throughout the revised manuscript. Specifically:

- **Font:** The manuscript has been formatted using a standard readable font (Times New Roman, size 12), avoiding the use of the “Symbol” font.
- **Headings:** All section and subsection headings have been clearly structured according to PLOS ONE’s three-level hierarchy (e.g., Introduction, Methods, Results, Discussion).
- **Footnotes:** All footnotes have been removed, and relevant information has been incorporated into the main text or references as appropriate.
- **Language:** The entire manuscript was rechecked for English grammar, clarity, and consistency to ensure compliance with the journal’s language standard.
- **Abbreviations:** All abbreviations are now defined at first mention in the text and used consistently thereafter. Non-standard or redundant abbreviations were removed.
- **References:** All references were reformatted according to the **Vancouver style**, following PLOS ONE and ICMJE guidelines. Author names, journal titles, volume/issue numbers, pages, DOIs, and URLs (where applicable) have been standardized.

We believe these comprehensive formatting updates ensure full compliance with the PLOS ONE style and presentation standards.
