# Supplementary material for: Economic evaluation of anti-malarial drug policies across presidential regimes in Nigeria: A comparative analysis from 1999 to present
Source: PLoS One. 2026 Mar 11;21(3):e0344909. doi: 10.1371/journal.pone.0344909 (PMC12978454; doi:10.1371/journal.pone.0344909)
Supplement: Attachment — Submitted filename: Reviewer_response_auresp_3.docx [file pone.0344909.s004.docx]

Reviewer #4 comment:

The revised manuscript looks much better. However, I still suggest the author to go through the submission guidelines (figure captions) very carefully and follow it appropriately.

Secondly, in the methods section there are lots of repetition and overlapping of words and sentences. For eg: The choice of perspective is mentioned in "Study Perspective" as well as in the "Evaluation Framework".

The author could maybe consider incorporating brief comments on future directions and call to action in the Conclusion section itself to improve clarity and conciseness. A seperate section may not be necesssary. Also the financial disclosure statement is not required in the manuscript file.

Author Response:

Dear Reviewer,

Thank you very much for your positive assessment of the revised manuscript and for your valuable additional suggestions.

In response to your comments, I have carefully reviewed the journal’s submission guidelines regarding figure captions and updated the manuscript accordingly. Figure captions are now positioned within the manuscript immediately after their first mention, in line with the journal’s formatting instructions, rather than being placed after the reference list as in the previous version.

Regarding the repetition and overlap identified in the Methods section, the manuscript has been revised to remove redundant wording and overlapping descriptions. Specifically, the repeated discussion of study perspective across the “Study Perspective” and “Evaluation Framework” sections has been streamlined to avoid duplication and improve clarity.

Additionally, the previously separate sections on Future Directions and Call to Action have now been incorporated into the Conclusion section to enhance clarity, conciseness, and narrative coherence, and the standalone sections have been removed accordingly. The Funding section has also been removed from the manuscript file as directed.

Thank you again for your helpful feedback, which has contributed to improving the clarity and overall quality of the manuscript.

Kind regards,

The Author

Reviewer #5 comment:

Authors have made the necessary changes.
All aspects have been addressed adequately.
Great work authors.
Sorry for the delay.
Thanks for the opportunity.

Author Response:

Dear Reviewer,

Thank you very much for your thoughtful evaluation of my revised manuscript. I sincerely appreciate your positive feedback and am pleased that the revisions have adequately addressed the concerns raised. Your constructive comments have been invaluable in improving the clarity and overall quality of the manuscript.

Thank you again for your time and for the opportunity to refine this work.

Kind regards,

The Author
